# Is metabolism the magic bullet for targeted cancer therapy?

**DOI:** 10.1186/s12885-023-10999-9

**Published:** 2023-05-30

**Authors:** Marija Trajkovic-Arsic, Elavarasan Subramani

**Affiliations:** 1 Division of Solid Tumor Translational Oncology, German Cancer Consortium (DKTK, Partner Site Essen) and German Cancer Research Center, DKFZ, Heidelberg, Germany; 2 West German Cancer Center, Bridge Institute of Experimental Tumor Therapy, University Hospital Essen, University of Duisburg-Essen, Essen, Germany; 3 Department of Cancer Systems Imaging, The University of Texas MD Anderson Cancer Center, Houston, TX USA

## Abstract

Altered cellular metabolism has long been recognized as a hallmark of cancer. Oncogenic signaling cascades induce metabolic rewiring that further supports tumorigenesis, therapy resistance and metastasis. In view of this, the Collection on ‘Cancer Metabolism’ highlights the current views and focus of research on personalized medicine approach to target metabolism for cancer therapy.

## Background

The last years of research have taught us that cancer is a very complex, multifaceted disease. Despite being “body-own”, cancer cells build up an aggressive, self-sustaining ecosystem with the aim of surviving, expanding and ultimately defeating the host. No matter how different the cancer from a normal cell, they rely on the same fundamental needs for nutrients and energy. While there are a few metabolic signatures that are unique to cancer cells and not found in normal cells, the metabolic processes are overall similar. The advantage of a cancer cell lies in its ability to rewire and adapt its metabolism to whatever source of food and energy is available. Activated oncogenes and signaling networks trigger alternative catabolic and anabolic processes that enable cancer cells to use substitute resources and improvise as needed.

The history of cancer metabolism started nearly a hundred years ago when Otto Warburg discovered aerobic glycolysis and lactate production in cultured tumor slices despite the availability of sufficient oxygen [1]. Warburg assumed that mitochondrial respiration is deficient in cancers for unknown reasons. Nowadays, we know that mitochondria are rarely damaged in cancers and genes coding for mitochondrial enzymes are highly preserved and rarely mutated, suggesting how essential and conserved respiration in cancers is. The activation of glycolysis as a process is nothing else but satisfying the need of a proliferating cancer cell for glycolytic inter-metabolites, ribose and hexose sugars that support nucleotide synthesis and protein glycosylation [2, 3]. Beyond glucose, cancer cells utilize alternative fuels to meet their energy demand which warrants further investigation for a complete characterization of cancer metabolism. Recent works emphasize that glycolysis is necessary for the regeneration of high NAD + levels needed for increased proliferation [4, 5]. Furthermore, cancer cells can scavenge the necessary nutrients from the microenvironment to promote the different steps of metastasis [6].

There are many factors to consider when performing and interpreting metabolic experiments. The broad variety of models ranging from in vitro 2D-, 3D-, co-culture cell systems to mouse models and even “in patient” in vivo approaches offer platforms for addressing many metabolic questions. Cell culture has been instrumental in addressing groundbreaking questions about which metabolic processes and nutrients are essential to maintain cancer growth. However, there are at least two significant drawbacks to cell culture systems which are especially discussed in metabolic terms. First, they lack microenvironmental support that is, as we now know, an indispensable part of the cancer ecosystem. Second, they rely on “non-physiological” concentrations of nutrients in the cell culture media. The standard cell culture media used world-wide were originally not designed to address metabolic questions but to support cell growth. Thinking in metabolic terms, feeding the cells with dramatically supraphysiological concentrations of glucose, glutamine and other essential nutrients may bias the metabolic findings and induce different responses to drug treatments. Performing metabolic experiments in media with nutrient concentrations that resemble more what is found in the tumor microenvironment may improve our views and understanding of what is metabolically really happening in the cancer cells. The recent introduction of “physiological” media [7, 8], is certainly a step forward in optimizing cell culture for metabolic research needs.

The cancer’s ability to rewire the metabolic pathways, adapt to the availability of nutrients and activate the non-canonical catabolic metabolism is its best adaptive fitness feature [9]. While metabolic rewiring is potentially the best support to uncontrolled proliferation, the different use of specific nutrients creates therapeutic vulnerabilities that can be targeted. Currently, there is a discussion on how a specific diet can influence the cancer metabolism and induce a metabolic dependency that can be targeted by keeping the cancer on a defined nutrient source. The most famous examples of dietary interventions in cancer are caloric restriction and the ketogenic diet, both showing varying levels of success and opposing effects in different cancer types [10, 11]. Recently, there has been a lot of attention focused on limiting dietary amino acids such as serine, glycine, methionine or glutamine, as their dietary removal has been shown to retard tumor growth in different mouse models [12, 13]. Additionally, there is recent evidence suggesting that gut microbiota-derived metabolites, such as indole-3-acetic acid, influence the response to chemotherapy in pancreatic ductal adenocarcinoma patients, further supporting the rationale for nutritional interventions during cancer treatment [14].

Due to the important role of metabolism in malignancy, metabolic imaging is now emerging as a powerful tool with the development of new radiotracers and MRI-based imaging agents that can provide real time signatures of cancer metabolism in both basic research and clinical settings. However, there has been a delay in translating these imaging approaches into effective methods for predicting and monitoring the response to cancer-targeted therapies. In the current era of precision medicine, such approaches would be invaluable in providing better information on treatment response and ultimately improving patient outcomes.

Finally, we would like to draw attention to the emerging subject of sexual dimorphism in cancer incidence and mortality, which highlights the genetic, epigenetic, hormonal, immune and metabolic differences between cancers in males and females [15]. Not surprisingly, the same cancer occurring in males or females may use different metabolic strategies and resources to survive. Thus, we need to acknowledge that the first step towards personalized oncology is to appreciate the patients’ sex and the metabolic specificities that it may bring.

In recognition of this important field, we are now welcoming submissions to our new Collection of articles titled ‘Cancer metabolism’. More details can be found here: https://www.biomedcentral.com/collections/CM.

Overall, a deeper understanding of metabolic changes that support cellular growth and function will open new horizons on how to utilize metabolism to fight against cancer.

## Data Availability

Not applicable.
